# Popular Pastimes as Aids to Hospital Funds

**Published:** 1890-02-01

**Authors:** A. W. Webster

**Affiliations:** An Assistant Secretary to the London Hospital Saturday Fund


### Popular Pastimes as Aids to Hospital Funds.

By A. W. Webster, An Assistant Secretary to the London Hospital Saturday Fund.

*(Continued from page 237.)*

### Lawn Tennis and Croquet.

We have now arrived at games in which females, by use and custom, figure as worthy partners to the sterner sex. Why they should not share our company in many more of our exercises I cannot quite understand. Physiologists, so far as I know, have never advanced any reasons why the female muscles should not be developed and strengthened in much the same manner as the male.

Prejudice in the matter of dress possibly accounts for female inactivity in athletic exercises, but surely when the sage wisdom of a large section of so august an assembly as that of the British Association condemns the corset and tight lacing in every shape and form, we may soon hope to see a reformation in female attire. I have already hinted that gymnastic exercises are becoming more popular in girls' seminaries. This training will have an influence on the future, and as a taste for more vigorous games is matured we shall find a demand for dress giving greater freedom of action, while we may safely trust the natural coquetry of the sex to provide nothing less graceful in style. Thus fashion may speedily accomplish what threatening doctors and lecturing scientists could hardly have brought into use by many years of patient labour.

Lawn tennis and croquet are doubtless games more practised by the upper and middle classes than by the working community, but this should not hinder exhibition games being played in aid of local charities.

Although I have heard of many very successful "lawn-tennis tournaments" being held in different parts of the country, I do not remember a single public exhibition in aid of a charity. Why should this be? The game itself does not lack interest either to players or spectators; indeed, it will allow of varieties of style and arrangement which few other athletic exercises can boast of. The possibility of mixing the "gentler" with the "sterner" sex, in "singles," "pairs," etc.; and the probability of seeing a "lord of creation" occasionally grovel in the dust before a graceful and agile conqueress are attractions not easily resisted by the public.

Most clubs are of a semi-private nature, and possess enclosed grounds. Let the officials of these show a little energy and enterprise, and display a determination to utilise these pleasurable pastimes for aiding good objects, and the public will soon appreciate their efforts and support their schemes.

### Open-Air Concerts and Garden Parties.

Favourable meteorological conditions being of the first importance in regard to the successful celebration of such events as are indicated in the above title, care should be taken in fixing dates to choose a time of year when we may reasonably hope for dry and genial weather to be the rule rather than the exception.

Really suitable accommodation will probably be difficult to obtain in many places. The well-kept lawn, the fine, grassy slope, the natural amphitheatre, the background of trees and shrubs, the sylvan retreats and shady nooks, and all other ideal arrangements are not often available near the great centres of population.

Practical enthusiasts in work of this kind will readily overcome mere difficulties of accommodation. Public parks, private suburban grounds, cricket or football fields, or, in fact, any well-situated piece of ground in an open part of the town can be made to serve the purpose of these gatherings.

Instrumental music is usually the chief item in the programmes of open-air concerts, but real success can only be attained by supplementing this with a variety of vocal pieces performed by artists of high, or, at any rate, popular, class. Most singers object to open-air performances, but the charitable object of the movement will frequently be found sufficient to overcome this obstacle, especially when the vocal part of the programme is confined to choruses, part pieces, or anything requiring the combined services of several performers at one time.

Amateur and sometimes professional dramatic performances have been held in the open air. The only example I can recall at the moment was the performance of a light comedy in a finely-wooded park in the South of England. Surrounding trees were utilised for fixing the most temporary yet effective scenery. The performance did not start till the dusk of a beautiful autumn day, when the row of lamps arranged as footlights showed to considerable advantage. The whole scene was novel, pleasant, and profitable. Although the accommodation was exceptionally good in this instance, everything might have been carried out under much less favourable circumstances, and the facilities available for ordinary open-air concerts may easily be utilised for these very similar entertainments.

The nature of accommodation must largely determine the style of entertainment at all open-air concerts, as it must also decide the advisability of charging for admission or the taking of a voluntary collection from visitors. My experience of the latter method in connection with charity concerts and other gatherings at which miscellaneous audiences assemble, enables me to place the average collection at less than threepence a head, and organisers of such entertainments will be safe in basing their calculations and anticipations on nothing higher, unless the circumstances be exceptional. The following quotation from a report in The Hospital will still further illustrate this point: "This age is nothing if not analytical. 10,571 separate coins were taken at the recent open-air concert held on behalf of the Wakefield Hospital. Of these 1 was a sovereign, 4 were half-sovereigns, 13 half-crowns, 20 florins, 153 shillings, 605 sixpences, 706 threepenny pieces, 6,764 pennies, 2,224 half-pennies, and one a postal order for a sovereign—if the last may be called a 'coin.' The estimated number of persons present was 15,000. Some of them evidently were there with the object of receiving charity, in the form of a concert, for nothing. Between four and five thousand, if not more, must have shirked the collection-boxes. Yorkshire, clearly, is not without its mean people."

Garden parties are luxuries usually confined to the higher and upper middle classes, but this should not prevent the happy owners of suitable grounds from occasionally inviting their friends and neighbours to a "charity levée" at which a collection might be taken in aid of a local hospital. An M.P. in one of the Midland counties recently carried out such an arrangement with much pleasure to his guests and great profit to a worthy charity.

The pleasures and practices of a garden party are totally unknown to the great mass of our working-class population, and wherever public parties have been held, the very novelty of the gatherings has always drawn large numbers of this and other classes together.

Will not some of our suburban landowners offer the use of their grounds now and again for this work and show their further interest by superintending the arrangements for the enjoyment of their guests?

In places where all attempts at securing picturesque and beautiful grounds have failed, I have known very successful gatherings held in an ordinary cricket ground, the ingenuity of the officials making ample amends for natural defects in scenery.

